# Sleep classification from wrist-worn accelerometer data using random forests

**DOI:** 10.1038/s41598-020-79217-x

**Published:** 2021-01-08

**Authors:** Kalaivani Sundararajan, Sonja Georgievska, Bart H. W. te Lindert, Philip R. Gehrman, Jennifer Ramautar, Diego R. Mazzotti, Séverine Sabia, Michael N. Weedon, Eus J. W. van Someren, Lars Ridder, Jian Wang, Vincent T. van Hees

**Affiliations:** 1 Netherlands eScience Center, Amsterdam, The Netherlands; 2 Department of Sleep and Cognition, Netherlands Institute for Neuroscience, Amsterdam, The Netherlands; 3 Perelman School of Medicine, University of Pennsylvania, Philadelphia, USA; 4 Divison of Medical Informatics, Department of Internal Medicine, University of Kansas Medical Center, Kansas City, KS 66160 USA; 5 Inserm U1153, EpiAgeing, Université de Paris, Paris, France; 6 Department of Epidemiology and Public Health, University College London, London, UK; 7 University of Exeter, Exeter, UK; 8 Eli Lilly and Company Ltd, Lilly Research Laboratories Neuroscience, Indianapolis, IN 46285 USA; 9 Accelting, Almere, The Netherlands

**Keywords:** Epidemiology, Sleep disorders, Neurophysiology

## Abstract

Accurate and low-cost sleep measurement tools are needed in both clinical and epidemiological research. To this end, wearable accelerometers are widely used as they are both low in price and provide reasonably accurate estimates of movement. Techniques to classify sleep from the high-resolution accelerometer data primarily rely on heuristic algorithms. In this paper, we explore the potential of detecting sleep using Random forests. Models were trained using data from three different studies where 134 adult participants (70 with sleep disorder and 64 good healthy sleepers) wore an accelerometer on their wrist during a one-night polysomnography recording in the clinic. The Random forests were able to distinguish sleep-wake states with an F1 score of 73.93% on a previously unseen test set of 24 participants. Detecting when the accelerometer is not worn was also successful using machine learning ($\hbox {F1-score} > 93.31\%$), and when combined with our sleep detection models on day-time data provide a sleep estimate that is correlated with self-reported habitual nap behaviour ($\hbox {r}=.60$). These Random forest models have been made open-source to aid further research. In line with literature, sleep stage classification turned out to be difficult using only accelerometer data.

## Introduction

Sleep quality and duration play an important role in human health^1^. Accurate methods for sleep assessment are needed to monitor the prevalence of poor sleep, to increase our understanding of the relation between sleep and health, and to design effective treatments for insomnia. Additionally, assessment methods need to have a high user-acceptability to reduce the risk of participant dropouts leading to selection bias.

The gold standard for sleep measurement, polysomnography (PSG), is prohibitively expensive and unfeasible for use in large scale population research. On the other hand, the much more feasible sleep diaries can provide information on time in bed but they are subject to recall bias and might be less relevant to assess time slept during this period. Therefore, wearable accelerometers have been explored since the mid-1990s as a possible alternative for multi-day real life (out of the lab) sleep monitoring.

To cope with memory and battery constraints in the early devices, data was pre-processed inside the device. Further, these devices had in common that they relied on piezo-electric acceleration sensors not sensitive to gravitational acceleration under static conditions. Technological advancements in the mid-2000s led to a new generation of accelerometers, referred to as raw data accelerometry, which was based on Micro Electro-Mechanical-Systems (MEMS) and able to store up to a week of digitised but otherwise unprocessed data in memory to facilitate offline analysis. These modern accelerometers are sensitive to gravitational acceleration under static conditions.

Offline access to raw data enabled revisiting the entire data processing pipeline as better algorithms emerge over time, which is needed to facilitate longitudinal studies sometimes spanning a lifetime. Further, access to raw data increased the ability to standardise analysis across studies to allow more meaningful comparisons. As a result, raw data accelerometry is now widely used by the health research community^2–4^.

Cole-Kripke^5^, Sadeh^6^, and Oakley^7^ proposed sleep detection algorithms for the accelerometer in the 1990s. Their algorithms had in common that data was pre-processed onboard the device towards a 30-second aggregate, called count. Cole and Sadeh derived counts with a zero-crossing technique, while Oakley derived counts with an amplitude-based technique. Cole, Sadeh, and Oakley, used a 7, 11, and 5 min time window for count-based sleep detection, respectively^5,6^.

Borazio *et al. *proposed the Estimation of Stationary Sleep-segments (ESS) algorithm for raw data accelerometry, which aims to detect segments of idleness quantified as a low standard deviation per second lasting for at least 10 min^8^. Next, van Hees et al.^9^ proposed an algorithm that relied on the estimated orientation angle of the accelerometer, based on the detection of time segments where the estimated angle of the accelerometer relative to gravity does not change beyond 5$^\circ$ for at least 5 min. This approach facilitated easier interpretation compared to the conventional approaches based on the magnitude of acceleration and zero-crossing counts. This heuristic algorithm is now extensively used in the research community^1,10–12^. More recently Trevenen et al. used machine learning to perform sleep classification. They extracted a variety of features from the acceleration vector magnitude and used these as input for a Hidden Markov Model (HMM) to classify sleep versus wakefulness, as well as to discriminate all four sleep stages^13^. The novel attempt to classify sleep stages from accelerometer-only data resulted in poor classification performance and was not able to accurately detect REM nor discriminate between Non-REM stages. Nonetheless, their conclusion about the potential for sleep stage classification was optimistic. Finally, Barouni and colleagues proposed a heuristic approach for sleep classification from raw data accelerometry, but mainly followed the approach used for traditional count-based accelerometers use kinematically hard to interpret the threshold crossing of the magnitude of acceleration^14^. Additionally, it should be noted that Willetts et al. used the term sleep classification in their work but relied on wearable cameras as criterion method. Wearable cameras are not able to distinguish sleep from wakefulness, by which their sleep detection claim is inaccurate^15^.

Complementary to sleep stage classification, information on day-time nap behaviour is also of interest. The assessment of nap behaviour is challenged by potential removal of the accelerometers for episodes during the day, since the existing heuristic nonwear detection algorithms^16^ was designed to only detect nonwear segments lasting for at least an hour.

Although the heuristic approaches have proven their value, their performance does in principle not improve when more data becomes available. In this paper, we explore the potential of random forests machine learning as a more data-driven approach to improve sleep-wake and wear-nonwear classification. Our approach uses data acquired from 158 participants from three different studies representing a wide age range and including both healthy sleepers and those with sleep disorders. The performance of these machine learning models was assessed by cross-validation using data from 134 participants (64 healthy sleepers and 70 with sleep disorders, age range 20–72)^9,17,18^. We then report the performance of our trained models on previously unseen test data from 24 remaining participants (16 healthy sleepers and 8 with sleep disorders). These trained models have been made open-source available to aid further sleep research. When used in combination, both sleep detection and nonwear detection approaches may be useful for daytime nap detection, which we evaluate in 109 separate individuals with accelerometer data collected in real life (out of the lab) where self-reported napping behaviour is available. Furthermore, we investigate the possibility of predicting four sleep stages (rapid eye movement (REM) sleep and Non-REM sleep stages N1, N2, and N3), which is not feasible with the current heuristic approaches. Reliable detection of sleep stages from wearable accelerometer data would advance sleep research as it provides an additional level of sleep description.

## Results

The number of samples corresponding to wakefulness and different sleep stages among the assessed 30 second intervals are shown in Table 1.

**Table 1:** Samples per class in the data (Percentages in parentheses).

Dataset	Nested Cross-validation	Test
Participants	134	24
Nonwear	264,880 (56.6%)	44,481 (54.1%)
Wake	35,355 (7.6%)	7041 (8.6%)
Sleep	167,969 (35.9%)	30,646 (37.3%)
N1	10,094 (2.2%)	1930 (2.3%)
N2	83,366 (17.8%)	15,439 (18.8%)
N3	41,729 (8.9%)	8097 (9.9%)
REM	32,780 (7%)	5180 (6.3%)
Total	468,204	821,68

### Sleep–wake classification

For sleep–wake classification, samples labeled as N1, N2, N3, and REM are considered as Sleep samples.

#### vanHees approach

The vanHees heuristic algorithm described in the “*Methods*” section is applied to the accelerometer data to obtain a binary classification of wakefulness or sleep. The classification performance of the method in the outer cross-validation and test set are reported in Table 2.

#### Random forests

**Figure 1:**
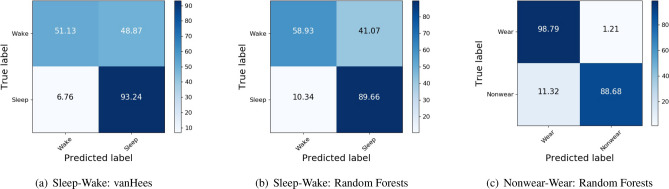
Sleep–wake and nonwear–wear classification on test set: confusion matrices for different methods. The numbers indicate the percentage of true labels that were predicted correctly or incorrectly.

The chosen hyperparameters for the trained random forests models for Sleep–Wake classification for each of the five cross-validation folds are given as (trees, max-depth) tuple—(400,Full), (500,Full), (400,Full), (200,Full), (200,Full) where max-depth of Full implies that the decision trees are allowed to grow to any depth till termination criteria are satisfied. The classification performance metrics across the five outer folds are averaged and reported in Table 2 along with test set performance. It can be observed that the random forests approach outperforms the vanHees approach on both outer cross-validation and test data. Specifically, random forests perform better at detecting wakefulness compared to the vanHees approach as seen in the confusion matrices in Fig. 1, though the number of sleep–Wake samples is heavily imbalanced.

The important features for sleep–wake classification averaged across all folds are shown in Supplementary Information Figure 4. For more information on feature definition see METHODS section. It can be observed that statistical measures for Locomotor Inactivity During Sleep (LIDS) and Z-angle are the most important features for sleep-wake classification.

**Table 2:** Binary sleep–wake and nonwear–wear classification.

Classification	Approach	Outer Cross-validation			Test		
		F1 (%)	AP (%)	Kappa	F1 (%)	AP (%)	Kappa
Sleep–wake	Sadeh	69.24	62.89	0.39	68.13	61.75	0.37
	Cole-Kripke	68.66	62.23	0.39	67.49	61.15	0.36
	vanHees	70.23	61.53	0.41	70.85	62.30	0.42
	Random forests	75.91 ± 2.43	80.27 ± 2.36	0.52 ± 0.05	73.93	78.76	0.50
Nonwear	Random forests	91.28 ± 2.31	96.95 ± 1.47	0.83 ± 0.05	93.31	99.08	0.85

*F1* F1-score, *AP* average precision (mean ± standard deviation).

#### Healthy versus poor sleepers

**Figure 2:**
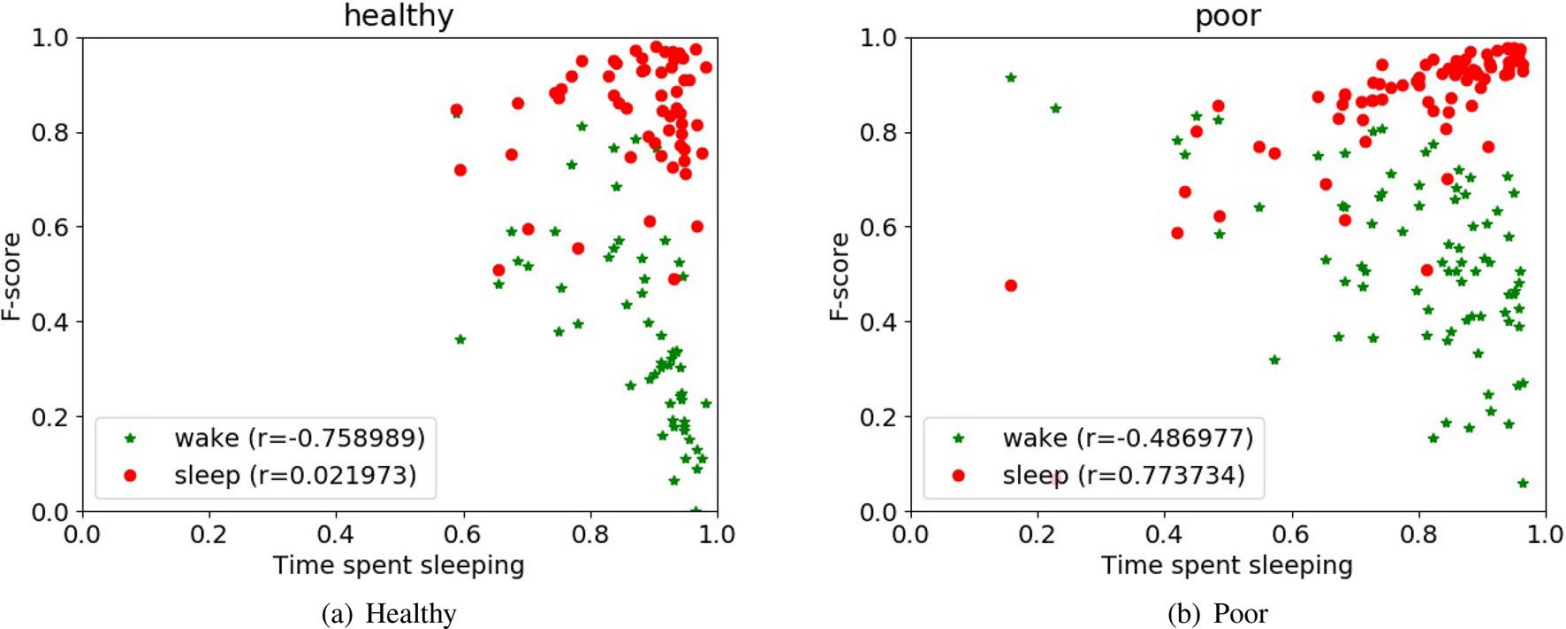
Sleep-wake performance random forests for healthy and poor sleepers (difference for wake and sleep is 11.28 ($\hbox {p}=0.0031$) and 5.57 ($\hbox {p}=0.0018$), respectively).

Figure 2 shows the plots of F1-score with respect to time spent sleeping for each user based on whether they are poor or healthy sleepers. Red markers denote F1-scores of sleep and green markers denote F1-scores of wakefulness for each user. We obtained the Spearman’s correlation coefficient for the time spent sleeping and F1-scores for healthy and poor sleepers. For healthy sleepers, it was observed that wakefulness F1-scores and time spent sleeping were negatively correlated due to fewer wake samples causing poor wakefulness classification. For poor sleepers, time spent sleeping was positively correlated with sleep F1-scores and negatively correlated with wakefulness F1-scores.

### Nonwear classification

The chosen hyperparameters for the trained random forests models for Nonwear classification for each of the five cross-validation folds are given as (trees, max-depth) tuple—(500,15), (100,15), (200,15), (300,20), (500,15). The nonwear classification performance metrics across the five outer folds are averaged and reported in Table 2 along with test set performance. It can be seen that nonwear classification using random forests performs quite well on both the outer cross-validation data and previously unseen test set.

The confusion matrices of nonwear classification are shown in Fig. 1 for the test set. The numbers in the matrices indicate the percentage of samples from the true class that was classified as the predicted class. It can be seen that Wear periods are predicted reliably whereas Nonwear periods tend to be confused with Wear periods by 11%.

The important features for nonwear classification averaged across all folds with random forests are shown in Supplementary Information Figure 7. It can be observed that statistical measures for LIDS and Z-angle are the most important features for nonwear classification.

### Sleep stage classification

Various classes in sleep classification can be organized into a hierarchy of classes as described in METHODS. Classifying accelerometer samples according to this hierarchy might help understand the discriminative properties (if any) of data to perform nonwear detection, sleep-wake, and sleep stage classification. Hence, we perform hierarchical classification of samples using the 36 engineered features and random forests.

The hierarchical classification performance metrics across the five outer folds are averaged and reported in Table 3 along with test set performance. Unlike F1-score computation of flat classification, F1-score of hierarchical classification takes all correctly classified ancestor classes of the hierarchy into account. It can be observed that the prediction performance drops as we go down the hierarchy. Levels 3, 4, and 5 which consist of leaf nodes like REM, N1, and N3 show a drastic reduction in performance.

**Table 3:** Hierarchical classification.

Hierarchy	Classes	Outer cross-validation		Test	
		F1 (%)	AP (%)	F1 (%)	AP (%)
Level 1	Nonwear	90.16	98.01	94.12	99.52
	Wear	89.22	96.15	93.79	99.09
Level 2	Wake	54.79	55.65	55.13	59.01
	Sleep	78.39	23.34	82.36	24.03
Level 3	NREM	64.55	21.34	72.02	20.19
	REM	16.67	14.96	12.27	16.18
Level 4	N1+N2	48.19	24.19	57.36	24.38
	N3	24.40	9.05	20.51	11.05
Level 5	N1	1.58	6.56	4.22	5.61
	N2	45.73	11.56	53.19	11.69

*F1* F1-score, *AP* average precision.

**Figure 3:**
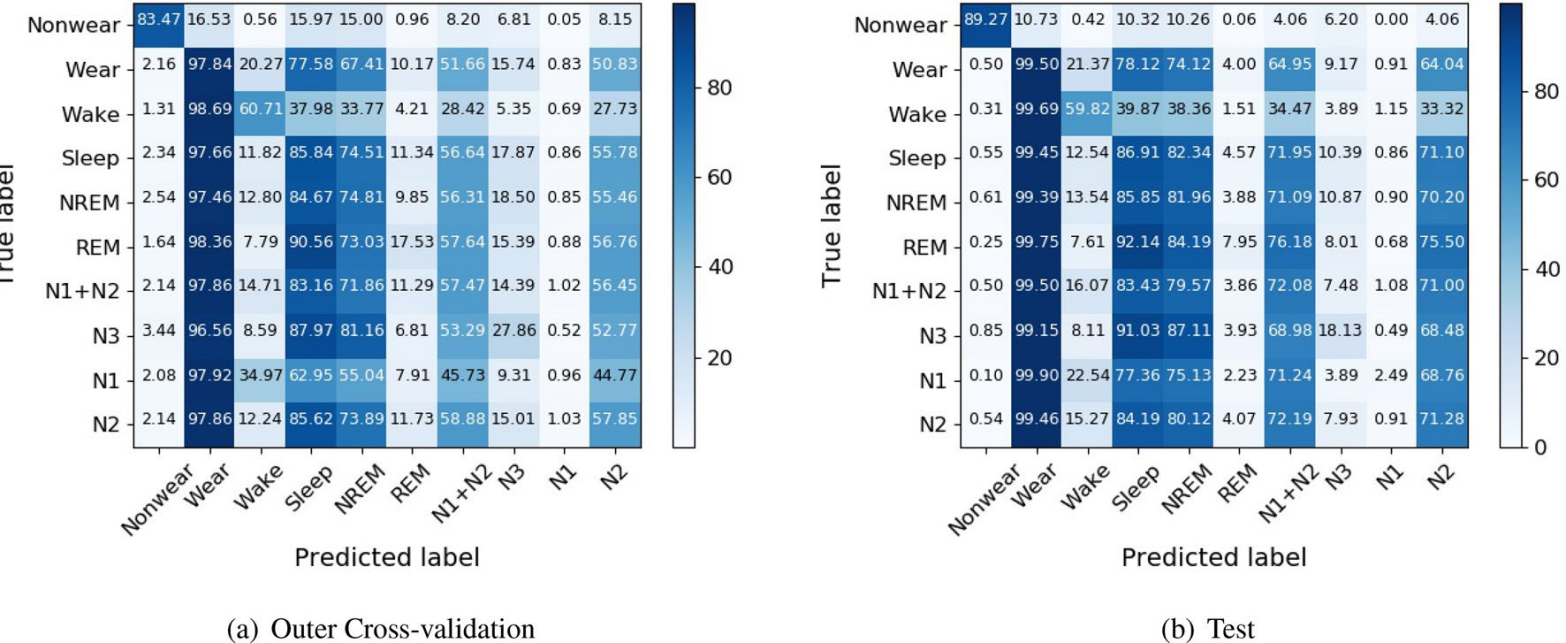
Confusion matrices for hierarchical classification.

The confusion matrices of true classes versus predicted classes are shown in Fig. 3. It can be observed that classes Wear, Sleep, NREM, N1 $+$ N2 and N2 seem to be predicted more frequently than other classes. The low diagonal values of REM, N3, and N1 show that it is difficult to discriminate between NREM & REM, N1 $+$ N2 & deep sleep (N3) and N1 & N2. Further, most samples classified as Sleep seem to be further classified as N2 which shows that N2 dominates the classification despite balancing the data with synthetic samples during training.

### Nap detection

Self-reported nap duration per week was 13 min less ($\hbox {t} = -0.36, \hbox {p} = 0.72$) compared with accelerometer-based estimates with a correlation of 0.60 ($\hbox {p} < .00001, \hbox {N}= 109$). A Figure of the corresponding data points can be found in the Supplementary information.

## Discussion

Based on our experiments, we infer that machine learning approaches such as random forests applied to accelerometer-only data improves the sleep–wake classification compared to the approaches proposed in 1990s^5,6^ and as well as the heuristic algorithm proposed by vanHees^9^. Our machine learning approach also enables nonwear detection at a higher time resolution than the vanHees approach^9^. The combination of these enhancements enables us to estimate daytime napping periods. However, the current findings should be seen as an encouragement of further research around nap detection and not as proof to justify immediate application in sleep research. Sleep stage classification from accelerometer data proved to be more challenging due to the absence of discriminative features in the data.

Our sleep classification approach is most similar to that of Trevenen et al.^13^. Based on an exploratory analysis of our data, we observed that transition between sleep states is rare compared to remaining in the same state for prolonged periods of time. Hence, when using approaches like Hidden Markov Models (HMM) as in Trevenen et al.^13^, the transition probability matrix is heavily diagonal and HMMs do not provide any advantage under such scenarios. Therefore, we trained discriminative models for every time interval based on engineered features. However, HMMs inherently ensure that the predicted sequences adhere to the transition probabilities and hence prevents spurious sleep state predictions. To have a similar effect for preventing spurious predictions, we smoothed the prediction probabilities of the individual random forest models over a 5-minute rolling window.

Further, while Trevenen et al. used data collected from healthy 22-year old individuals, our experiments are based on a more challenging, yet more heterogeneous dataset collected from a wide range of ages and includes participants with sleep disorders.

We explored random forest-based nonwear detection to gain insight into the potential for daytime nap detection. Nonwear detection was found to be acceptably accurate. By feeding the classifier both sleep and nonwear data we offered the classifier a challenging task. If we had trained it using data corresponding to nonwear and a person performing activities the classification task would have been easy but not representative of real-life nonwear detection. Future research is needed to identify generic purpose non-wear detection able to both assist in the distinction of daytime naps and the identification of large episodes of nonwear or sleep.

Sleep stage classification was expected to be challenging at the outset of the study. However, the reason why we still explored it is that even a weak classification could be of value in large scale population studies, e.g. UK Biobank^10^, where minor effects become only visible when averaged over a large number of individuals. Sleep stage classification may only be realistic with complementary sensor data, e.g. photoplethysmogram (PPG), which was outside the scope of this study as we focus on solutions for the already widely collected accelerometer-only data.

The positive correlation of 0.60 and the lack of a statistical difference between average self-reported habitual napping duration and estimates from our ensemble of accelerometer-based random forest models are encouraging. However, based on these data alone it is hard to say whether the observed individual differences are explained by the subjective nature of a questionnaire, the discrepancy between the questionnaire that asks about habitual behavior and an accelerometer recording corresponding to nine specific days, or the precision of the random forest models. Therefore, further research is warranted involving a more direct comparison, e.g. with video observation.

Most of the data used in this study was collected with the GENEActiv accelerometer brand. Future studies should consider the potential of model transferability across accelerometer brands. Previous research indicates that data is highly comparable across accelerometer brands^19^, but confirmation of these specific outcomes is desired.

Models were trained and tested across three different datasets. The PSG data was scored by a different sleep technician at every site, each site had its own PSG equipment, and participants at each site had different demographics. It could be hypothesized that the models have therefore become more robust against signal artifacts related to these experimental differences.

The present study does not look at detecting the beginning and the end of the night (sleep period time window), which is a different but related challenge we looked at in van Hees et al.^20^.

Raw data accelerometry faces the same challenges as traditional actigraphy in not being able to capture most physiological processes that underlie sleep. Our present work does not prove, or even attempt to prove, that raw data offers more accurate sleep detection than traditional actigraphy. The main advantage of raw data is that it offers increased scientific transparency and can be re-processed for many purposes beyond sleep research alone.

Our work shows that random forests can help to enhance the sleep classification relative to the currently open-source available method by van Hees et al.^9^, and the Sadeh and Cole-Kripke implementation by Hammad *et al. *^21^. Sleep researchers will have to decide whether they prefer a more accurate but less interpretable random forest model or a less accurate model by vanHees, Sadeh, or Cole-Kripke. In an earlier publication, we argue that the vanHees heuristic model is more kinematically interpretable compared with conventional algorithms that rely on zero-crossing counts or magnitude of acceleration^9^. Whether the Sadeh and Cole-Kripke algorithms offer better methodological consistency with historical research is difficult to say since the piezo-electric acceleration sensors as used in the 1990s have been replaced by MEMS-based capacitive sensors in the 2000s that have a wider frequency response. We are not aware of any studies that investigate the comparability of Sadeh or Cole-Kripke algorithm output across these hardware generations.

There are also important clinical implications of these results. The assessment of sleep/wake patterns for the diagnosis of sleep and circadian rhythm disorders often requires polysomnography, which is expensive and labor-intensive. Accelerometry is sometimes used as a less expensive form of assessment but current algorithms are limited in their accuracy, particularly in patients with insomnia. Improved algorithms have the potential to make accelerometry a more clinically-useful assessment tool that would permit the measurement of sleep and wake over extended periods of time. This approach could also be implemented more easily than polysomnography in non-sleep clinic settings. Future studies are warranted to investigate the physiology behind misclassifications in order to better understand how sleep classifier performance may vary across specific sleep disorders.

## Methods

The raw accelerometer data was extracted from binary files obtained with different accelerometer brands using R package GGIR^22^. The raw data was then preprocessed using GGIR algorithms for signal calibration relative to gravitational acceleration^23^ and alignment of PSG assessment labels with processed data. Next, we explored random forests machine learning to perform sleep-wake, nonwear-wear, and sleep stage classification. Additionally, we explored the value of sleep-wake and nonwear-wear classification to identify daytime naps.

### Random forests

The same random forests approach was used for sleep-wake, nonwear-wear, and sleep stage classification. In our initial exploration of the data, we experimented with deep learning techniques but as the results were not better than the vanHees approach we decided to report them in the Supplementary  Information to this paper.

#### Signal features

In our models, we used 36-dimensional features encompassing twelve different statistical measures, listed in Table 4, applied to three derived signals calculated from the three accelerometer axes, $a_x, a_y,$ and $a_z$:

- *ENMO* : The Euclidean Norm Minus One (ENMO) with negative values rounded to zero in *g* has been shown to correlate with the magnitude of acceleration and human energy expenditure^16^. ENMO is computed as follows:
  1 $$\begin{aligned} ENMO = max(0, \sqrt{a_x^2 + a_y^2 +a_z^2} - 1) \end{aligned}$$
- *Z-angle*: Z-angle, computed using Eq. 3, corresponds to the angle between the accelerometer axis perpendicular to the skin surface and the horizontal plane. As described in “vanHees approach”, any change (or lack of change) in the z-angle over successive time intervals may be an indicator of posture change.
- *LIDS*: Locomotor Inactivity During Sleep (LIDS)^24^ involves a non-linear conversion of locomotor activity and has shown to be sensitive to ultradian sleep cycles. The original paper did not make use of raw data accelerometry. In this work, LIDS is computed as follows:
  2 $$\begin{aligned} LIDS = \frac{100}{activity\ count + 1} \end{aligned}$$
  where $activity\ count$ is computed using a 10-minute moving sum over $max(0,ENMO - 0.02)$. LIDS is then smoothed using moving average over a 30-min window.

For each 30 s interval, we computed 36-dimensional features which were then used to train the random forest.

**Table 4:** Statistical measures applied to derived signals.

Statistical measure	Description
Mean	Mean value of the signal in that interval
Std	Standard deviation of the signal in that interval
Minimum	Minimum value of the signal in that interval
Maximum	Maximum value of the signal in that interval
MAD	Median absolute deviation of the signal in that interval
Entropy20	Entropy of the signal at low resolution (20 bins)
Entropy200	Entropy of the signal at high resolution (200 bins)
Prev30Diff	Difference in mean value between the previous 30 s and current interval
Next30Diff	Difference in mean value between the current interval and next 30 s
Prev60Diff	Difference in mean value between the previous 60 s and current interval
Next60Diff	Difference in mean value between the current interval and next 60 s
Prev120Diff	Difference in mean value between the previous 120 s and current interval
Next120Diff	Difference in mean value between the current interval and next 120 s

#### Imbalanced data

We observed from our data that some labels occur more frequently than others leading to an imbalanced dataset. Such data needs to be handled with care when used with machine learning models since the model might learn to always predict the class with the majority of samples. A typical workaround is to undersample or oversample the training samples belonging to various classes such that the model is trained with roughly equal number of samples from each class. In this paper, we followed oversampling of classes using Synthetic Minority Over-sampling Technique (SMOTE)^25^. SMOTE generates new samples by interpolation of random samples with their nearest neighbors. In our work, we used the SMOTE implementation in the *imbalanced-learn* python package^26^ with a sampling strategy to resample all classes to have roughly equal number of training samples.

#### Performance metrics

As our data is heavily imbalanced, the classification performance of our experiments was evaluated using F1-score and Average Precision, *i.e. *area under the Precision-Recall curve. Note that SMOTE was only applied to the training data, this why we still need to account for data imbalance in the performance evaluation. F1-score is the harmonic mean of precision and recall with high F1-scores indicating good classification performance. F1-scores of individual classes are averaged to obtain the overall F1-score, *i.e. *macro-averaging, to treat all classes equally. Additionally, we report Cohen’s Weighted Kappa coefficient for Sleep-wake classification results to facilitate comparisons with other studies^27^.

F1-scores are computed using predicted classes chosen with specific thresholds. However, the precision-recall curve gives a better picture of the classification performance since it plots recall vs precision by varying thresholds. Better classification performance is indicated by curves tending towards the top right. The area under the precision-recall curve, *i.e. *Average Precision, gives a quantifiable measure of performance with Average Precision of 1 indicating best performance.

#### Training and evaluation

The resampled features were used to train random forests models^28^. Classification using Random forests works by training multiple decision trees with subsets of the data and averaging the decision tree outputs to address overfitting. The features were normalized to have zero mean and unit standard deviation before training.

We used a nested cross-validation approach, involving: fivefold inner cross-validation to optimise hyper parameters, and a fivefold outer cross-validation to obtain generalisation performance. This means that 5 $\times$ 5 models were trained in the process, out of which five models from the outer cross-validation can be used as an ensemble on new data.

In the inner cross-validation, a randomized hyperparameter search was used to choose the number of trees from (100, 150, 200, 300, 400, 500) and the tree depth from (5, 10, 15, 20, Full). Other random forests parameters were retained as default as specified by the scikit-learn package. Each inner cross-validation fold splits the training data into training and validation data (4:1) where the validation data is used to choose the optimal hyperparameters *i.e. *number of trees and tree depth, for each fold based on Average Precision. Hence, each inner cross-validation fold will use a different random forests model tuned optimally for its corresponding training data. The outer cross-validation is used to obtain both F1-scores and Average Precision (AP) for generalization performance. For the outer cross-validation, the data is split into training and validation partitions such that participants in both partitions do not overlap. This ensures that the algorithm does not learn any patterns specific to participant behavior. To ensure that the output is not spurious, we smoothed the prediction probabilities of the individual random forest models over a 5-min rolling window before computing performance metrics. Finally, a left out test set with 24 individuals is used to obtain the generalisation performance of the ensemble of the models generated in the outer cross-validation using averaged prediction probabilities. The same ensemble of models is used in “*Nap detection*”.

### Sleep–wake classification

In order to benchmark the performance of our models for sleep-wake classification, we used the previously published vanHees approach^20^ as baseline. In addition, we also used the implementations^21^ of Sadeh^6^ and Cole-Kripke^5^ approaches for comparison. Both these approaches use aggregated actigraphy counts with a zero-crossing technique to perform sleep–wake classification.

#### vanHees approach

To estimate sleep, van Hees et al.^9^ proposed a heuristic algorithm using accelerometer data. This algorithm uses (lower) arm angle relative to the gravitational component estimated from accelerometer data to differentiate between sleep and wakefulness states. The arm angle is estimated as:

3 $$\begin{aligned} angle_z = tan^{-1}\left( \frac{a_z}{\sqrt{a_x^2 + a_y^2}}\right) .\frac{180}{\pi } \end{aligned}$$

where, $a_x, a_y,$ and $a_z$ are median values of the three accelerometer axes computed over a rolling five-second window. The vanHees algorithm performs the following steps to distinguish between Sleep and Wake states:

1. Average Z-angles for every 5 s.
2. Identify the time window where the angle does not change by more than 5 $^{\circ }$ for at least 5 min.
3. Label corresponding time windows as sleep.

#### Healthy versus poor sleepers

We analyzed the sleep–wake classification based on the health state of the participants. Poor sleepers are those participants who have been diagnosed with various sleep disorders while healthy sleepers are those without any sleep disorders.

### Nonwear detection

Periods of nonwear less than 30 min will go undetected with available heuristic approaches^14,16^. We investigated whether nonwear periods can be determined at a higher resolution with machine learning (random forests). The ground truth labels for our nonwear classification are defined based on two assumptions: The accelerometer is worn during the PSG recording as prescribed by the study protocol and supervised by the researcher, and the accelerometer is not worn outside the PSG recording, according to the study protocol. Only if the standard deviation in the acceleration signal per 15 min is larger than 13.0 mg (1 mg = 0.00981 m/s$^2$) these 15 min outside the PSG recording are labelled as wear. Here, the threshold of 13.0 mg is borrowed from the Heuristic van Hees approach.

### Sleep stage classification

**Figure 4:**
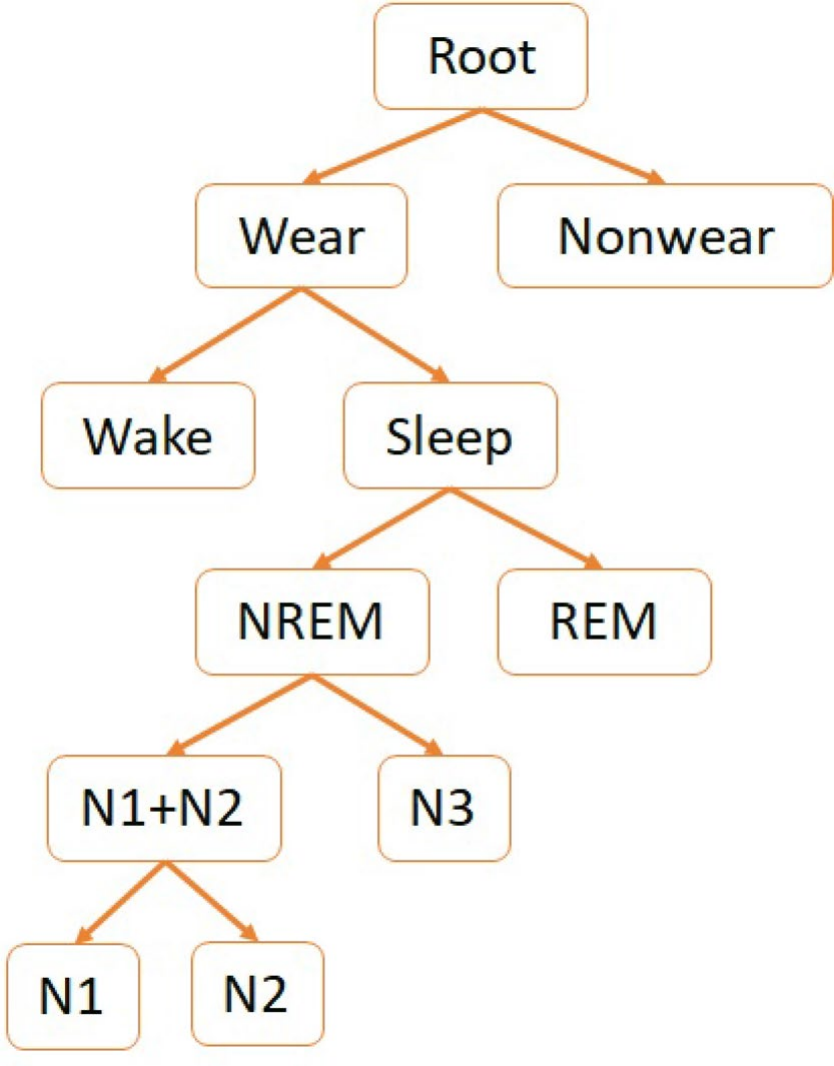
Hierarchy of classes used in sleep stage classification.

The various stages in sleep classification can be organized into a hierarchy of classes as shown in Fig. 4. These follow the standard neurobiological definitions of sleep. We grouped N1 and N2 because they are more similar than N2 and N3, particularly from an electrophysiological perspective (e.g., EEG and EMG). Classifying accelerometer samples according to this hierarchy might help understand the discriminative properties (if any) of the data to perform nonwear detection, sleep–wake and sleep stage classification. Hence, we perform hierarchical classification of samples using random forests as described in “*Random forests*”.

For hierarchical classification, we trained a random forest model for every non-leaf node to classify samples into one of its child nodes. Since the samples belonging to each node are imbalanced, we balanced the training samples for each non-leaf node using SMOTE with the sklearnhierarchicalclassification implementation.

### Nap detection

We combined the random forest models for sleep–wake and wear-nonwear classification as presented in this paper to distinguish: Nonwear, Sleep, and Wake, and applied these to real-life (out of the lab) the accelerometer data. Total weekly napping time was calculated as the total duration of all classified sleep episodes that last at least 15 minutes and are outside the Sleep Period Time Window. Here, Sleep Period Time Window was guided by the available sleep log. A t-test, Pearson’s correlation coefficient and scatter plot are used to inspect the relation. The data from 109 individuals as used are a sub-sample of the Whitehall II Study data^9^ over-sampled with individuals who report nap behaviour, detailed information on the data and sampling can be found in the Supplementary information.

### Ethical approval and informed consent

The studies were approved by the University College London ethics committee (85/0938), NRES Committee North East Sunderland ethics committee (12/NE/0406), University of Pennsylvania ethics committee (819591), and VU University Medical Center Amsterdam, respectively. Methods reported in this manuscript were performed in accordance with relevant guidelines and regulations covered by the aforementioned ethics approval committees. All participants provided informed consent.

## Supplementary Information

Supplementary Information.

## Data Availability

The classification models developed in this paper are available as open access data on Zenodo^29^. The R^30^ package GGIR was previously developed for the processing of accelerometer data^22^. We enhanced GGIR to be able to embed the sleep classification models written in Python as explained in the GGIRpackageVignette^31^. Specific code to use this functionality in combination with the models from this paper can be found here. The combination of the code and GGIR package allow for sleep classification and nonwear classification of raw accelerometer data. This involves data extraction, pre-processing, feature extraction, and sleep or nonwear classification. Raw data from the polysomnography study in Newcastle has been made open access available in anonymized format on zenodo.org^32^. Data from the University of Pennsylvania are available through the National Institute of Mental Health data archive. Whitehall II data, protocols, and other metadata are available to the scientific community. Please refer to the Whitehall II data sharing policy which can be found here.
