# Supplementary material for: Sleep classification from wrist-worn accelerometer data using random forests
Source: Sci Rep. 2021 Jan 8;11:24. doi: 10.1038/s41598-020-79217-x (PMC7794504; doi:10.1038/s41598-020-79217-x)
Supplement: Supplementary file 1 — Supplementary Information. [file 41598_2020_79217_MOESM1_ESM.pdf]

Supplementary Information for Article: Sleep classification from wrist-worn accelerometer data using Random Forests

Kalaivani Sundararajan[1], Sonja Georgievska[1], Bart H. W. te Lindert[2], Philip R. Gehrman[3], Jennifer Ramautar[2], Diego R. Mazzotti[4], Séverine Sabia[5,6], Michael N. Weedon[7], Eus J. W. van Someren[2], Lars Ridder[1], Jian Wang[8], and Vincent T. van Hees[1,9,*]

[1]Netherlands eScience Center, Amsterdam, The Netherlands
[2]Department of Sleep and Cognition, Netherlands Institute for Neuroscience, Amsterdam, The Netherlands
[3]Perelman School of Medicine of the University of Pennsylvania, USA
[4]Divison of Medical Informatics, Department of Internal Medicine, University of Kansas Medical Center, Kansas City, KS, 66160, USA
[5]Université de Paris, Inserm U1153, EpiAgeing, France
[6]Department of epidemiology and public health, University College London, London, UK
[7]University of Exeter, Exeter, United Kingdom
[8]Eli Lilly and Company Ltd, Lilly Research Laboratories Neuroscience, Indianapolis, IN 46285, USA
[9]Accelting, Almere, The Netherlands
[*]v.vanhees@accelting.com

1 Preprocessing

The raw accelerometer data was extracted from binary files corresponding to different accelerometer brands using R[1] package GGIR[2,3]. However, it needs to be processed before it can be used for classification. The preprocessing of the raw data, also done with GGIR, involves signal calibration relative to gravitational acceleration and nonwear detection. The alignment of polysomnography (PSG) assessment labels with raw data was done with custom Python scripts[4].

Calibration

An accelerometer converts acceleration into an electrical signal using a linear relationship characterized by a gain factor and an offset. Sensor calibration involves estimation of these parameters. Typically, accelerometers are calibrated by the manufacturers and they can be validated manually to correct any errors. However, when data has been collected in the past and the measurement device no longer exists it is impossible to conduct new validation experiments. Therefore, auto-calibration techniques[5] have been proposed which allow estimation of calibration parameters from accelerometer data of the participant without any additional experiments. The technique involves monitoring observed accelerometer data for periods of non-movement. A moving average is computed over these periods from each of the three orthogonal sensor axes to generate a three-dimensional ellipsoid representation. In an ideal state, this representation would be a sphere with radius $1g$. Hence, the deviations between the radius $1g$ of the sphere and the radius of the three-dimensional ellipsoid are used to derive correction factors to rectify axis-specific calibration errors[5].

Label assignment

The classification of sleep stages within PSG is typically done by a sleep technician who analyzes every few seconds of various EEG signals to assign the corresponding sleep stage. In this paper, we use these assigned sleep stages as ground truth labels in our machine learning models for accelerometer data. Next, we aligned these labels with accelerometer data by matching PSG timestamps with that of accelerometer data. In our datasets (see Section *Datasets*), sleep stages are estimated for every 30 seconds of night-time sleep, ranging from 10PM to 7AM. The PSG technician labels every 30 seconds as Wake, NREM1, NREM2, NREM3 or REM state. For sleep-wake classification, we considered NREM1, NREM2, NREM3 and REM as Sleep.

Example data

An example of preprocessed accelerometer data over one night is shown in Figure 1 with sleep stages estimated using PSG (gold standard criterion) and nonwear periods detected based on study protocol as described in the main manuscript. Time intervals outside the PSG experiment and not detected as nonwear are unlabeled.

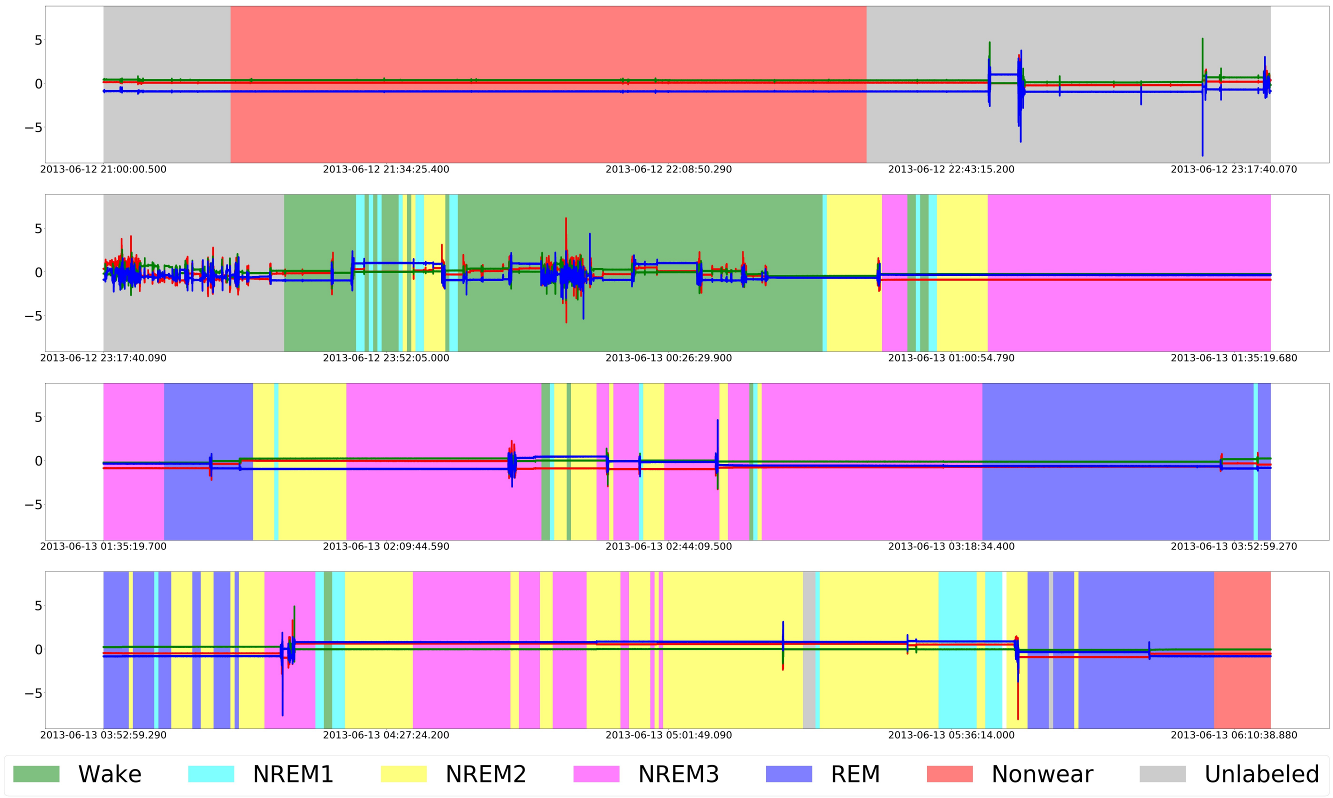

Figure 1. An example of a participant's preprocessed accelerometer data with polysomnography (PSG) derived sleep stages and heuristically detected nonwear periods. Each horizontal block of data represents 2 hours and 15 minutes of accelerometer data. Red, green and blue signals indicate the three axes (a_x, a_y, a_z) of the accelerometer.

2 Datasets

We used three datasets collected from both poor and healthy good sleepers in our experiments. The datasets include raw accelerometer data collected for one or two nights and polysomnography (PSG) assessment over that period. The PSG assessments, made every 30 seconds, are used as gold-standard labels for different sleep stages in our experiments.

Newcastle

The Newcastle dataset[6] consists of accelerometer data collected from 28 sleep clinic patients scheduled for polysomnography (PSG) assessment at the Freeman Hospital, Newcastle upon Tyne, UK. The sleep clinic patients were aged between 21 and 72 years (mean±sd: 45±15 years) with sleep disorders such as hypersomnia, insomnia, REM behaviour disorder, sleep apnoea, narcolepsy, sleep apnoea, parasomnia, restless leg syndrome, sleep paralysis, and nocturnia. The accelerometer data and PSG assessment were collected for one night. All 28 patients had data collected for the left wrist while only 27 patients had data collected for the right wrist. For a more detailed description see[7].

Pennsylvania

The Pennsylvania dataset[8] consists of data from 22 healthy sleepers scheduled for PSG assessment at the University of Pennsylvania Center for Sleep. The participants were aged between 18 and 35 years (mean±sd: 22.8±4.5 years). The accelerometer data and PSG assessment were collected for one night from the non-dominant wrist of all participants. For a more detailed description see[9].

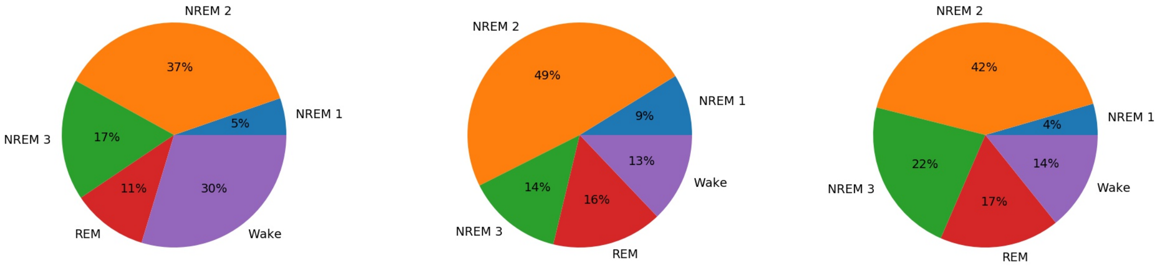

(a) Newcastle (b) Pennsylvania (c) Amsterdam

Figure 2. Wakefulness and sleep stage distributions in the datasets

Table 1. Samples per class in the datasets

Dataset	Nonwear	Wake	Sleep	NREM 1	NREM 2	NREM 3	REM	Total
Newcastle	12974	16044	38013	2868	19827	9428	5890	67031
Pennsylvania	166473	2290	15480	1560	8644	2451	2825	184243
Amsterdam	128866	24062	145122	7596	70334	37947	29245	298050

Amsterdam

The Amsterdam dataset consists of data collected from 114 individuals recruited by the VU University Medical Center, Amsterdam, The Netherlands[10]. The participants were screened using online questionnaires and telephone interviews resulting in two groups of participants based on Insomnia Severity Index (ISI). The group with insomnia disorder (ISI $\geq$ 10) consists of 58 participants aged 21 to 69 years. The other group consists of 56 healthy sleepers (ISI < 10) aged 22 to 70 years. Accelerometer data and PSG assessment were collected for one or two nights.

The wakefulness and sleep stage distributions among the assessed 30 second intervals for all three datasets are shown in Figure 2. Table 1 shows the number of samples per class for all three datasets.

For our experiments, the above three datasets are pooled and split into Train and Test partitions to train and evaluate our machine learning models. The demographics of both partitions are shown in Table 2 based on users who have this data reported. The wakefulness and sleep stage distributions for Train and Test partitions are shown in Figure 3. Table 3 shows the number of samples per class for both partitions.

2.1 Nap detection in Whitehall II Study, United Kingdom

To gain better insight in the potential of nap detection we looked at data from the Whitehall II Study in the United Kingdom[7]. Participants were asked to fill in a Stress and Health survey[11] and were then asked to wear an accelerometer on their wrist for nine days. Question 57 of the Stress and Health[11] survey asks the participant about their napping behavior: "Do you doze or take a nap anytime during the day or behfore you go to bed?", and if answered with Yes the participant is asked to indicate the frequency and average duration of the naps based on 4 and 5 Likert scale, respectively. From this habitual weekly napping duration can be estimated by multiplying the frequency and average duration of naps. Out of the 4021 individuals for with both questionnaire data and at least two week- and two weekend days with accelerometer data for at least 16 hours per day we took a random sample of 20 individuals who do not report to take naps. Additionally we took 10 random individuals from each of the four nap frequency categories and each of the five nap duration categories. Age: 69.7 years (Inter quartile range: 64-74). Body Mass Index: 26.35 kg/m^2 (Inter quartile range: 23.6-28.7). From these 20 individual reported to take no naps,

Table 2. Demographics of the Train and Test partitions

Partition	Healthy	Poor	Male	Female	Age
Train	64	70	49	82	18-72
Test	16	8	12	12	19-68

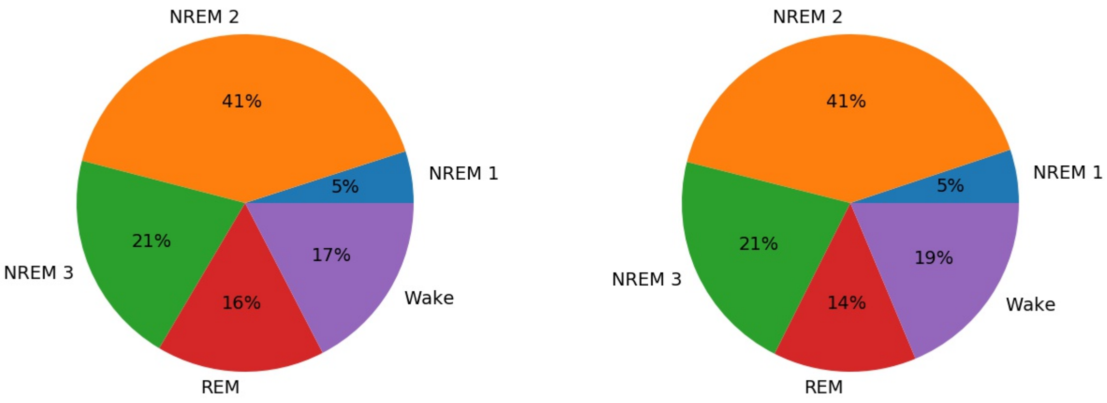

(a) Train (b) Test

Figure 3. Wakefulness and sleep stage distributions in the Train and Test partitions

Table 3. Samples per class in the Train and Test partitions

Partition	Nonwear	Wake	Sleep	NREM 1	NREM 2	NREM 3	REM	Total
Train	264800	35355	167969	10094	83366	41729	32780	468124
Test	44481	7041	30646	1930	15439	8097	5180	82168

and the distribution across the four nap frequency categories was: 21, 27, 29, and 12. The distribution across the four duration categories was: 32, 24, 13, 10, and 10.

3 Sleep-Wake classification

Performance scores aggregated by user

The performance scores for Sleep-Wake classification, F1-score, Average Precision and Kappa score, were averaged over users (unlike over cross-validation folds in the main paper) and the results are reported in Table 4.

Precision-Recall curves

The precision-recall curves for Wake and Sleep classes are shown in Figures 4 and 5.

Feature importances

The important features for Sleep-Wake classification are shown in Figure 6.

Table 4. Binary Sleep-Wake classification aggregated by user
F1 - F1-score, AP - Average Precision

Approach	Outer Cross-validation			Test		
	F1 (%)	AP (%)	Kappa	F1 (%)	AP (%)	Kappa
Sadeh	65.15 ± 10.51	61.72 ± 7.78	0.33 ± 0.18	66.56 ± 13.38	63.10 ± 9.92	0.36 ± 0.24
Cole-Kripke	64.82 ± 10.50	61.29 ± 7.71	0.33 ± 0.18	66.29 ± 13.07	62.75 ± 9.74	0.36 ± 0.23
vanHees	65.95 ± 9.91	60.01 ± 8.15	0.33 ± 0.19	67.90 ± 11.36	62.20 ± 9.64	0.37 ± 0.22
Random Forests	71.09 ± 11.41	77.69 ± 11.95	0.44 ± 0.21	70.93 ± 11.25	79.21 ± 11.29	0.43 ± 0.21

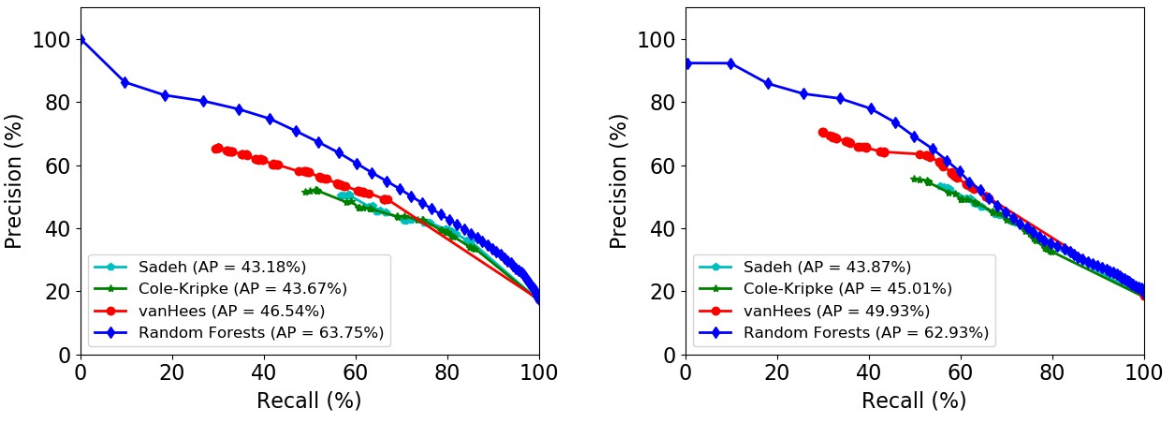

(a) Outer Cross-validation

(b) Test

Figure 4. Sleep-Wake classification: Precision-Recall curves for Wake

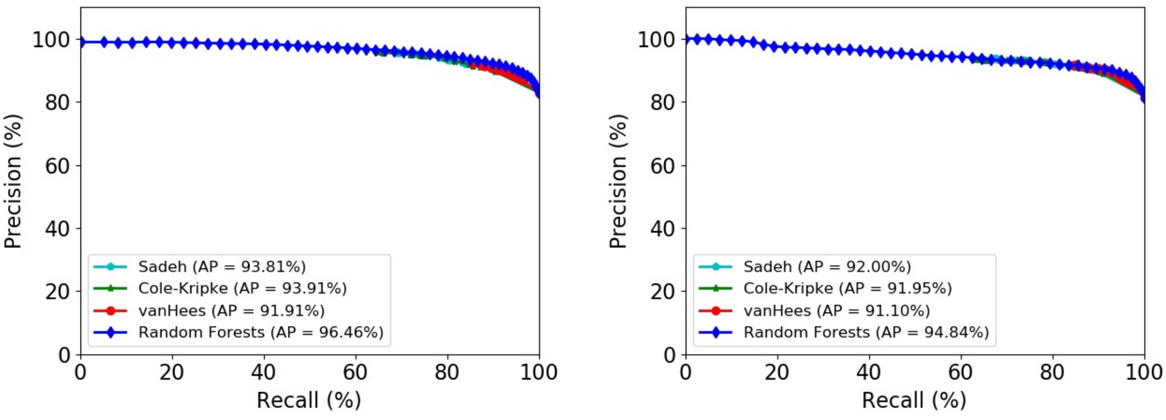

(a) Outer Cross-validation

(b) Test

Figure 5. Sleep-Wake classification: Precision-Recall curves for Sleep

4 Nonwear classification

Performance scores aggregated by user

The performance scores for Nonwear-Wear classification, F1-score, Average Precision and Kappa score, were averaged over users and the results are reported in Table 5.

Precision-Recall curves

The precision-recall curves for Nonwear and Wear classes are shown in Figures 7 and 8.

Feature importances

The important features for nonwear classification are shown in Figure 9.

5 Sleep stage classification

The sleep stage classification performance metrics across the five outer folds are averaged and reported in Table 6. Similarly, the precision-recall curves for five classes are shown in Figures 10, 11, 12, 13 and 14. The important features for sleep stage classification averaged across all folds are shown in Figure 16. It can be observed that statistical measures for LIDS and Z-angle are the most important features for sleep stage classification across all three datasets.

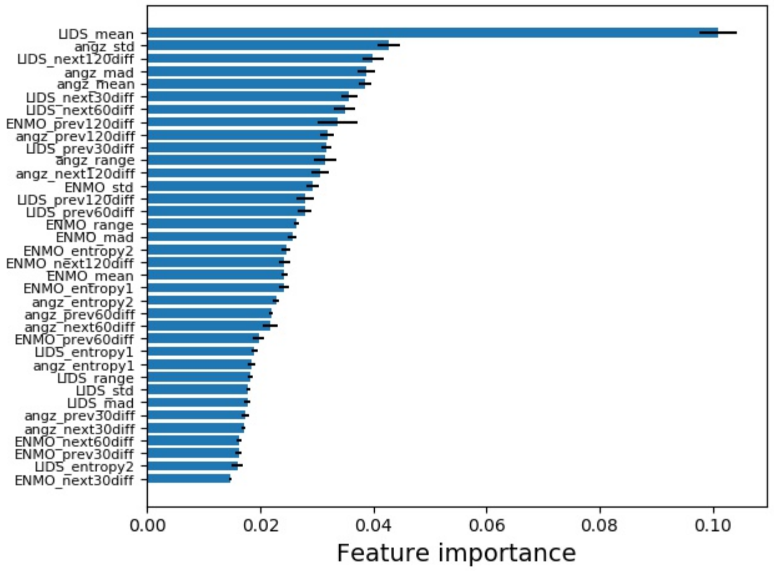

Figure 6. Random Forests feature importances for binary classification

Table 5. Binary Nonwear-Wear classification aggregated by user
F1 - F1-score, AP - Average Precision

Approach	Outer Cross-validation			Test		
	F1 (%)	AP (%)	Kappa	F1 (%)	AP (%)	Kappa
Random Forests	83.66 ± 11.35	90.29 ± 10.03	0.68 ± 0.21	81.77 ± 13.15	91.57 ± 12.38	0.64 ± 0.26

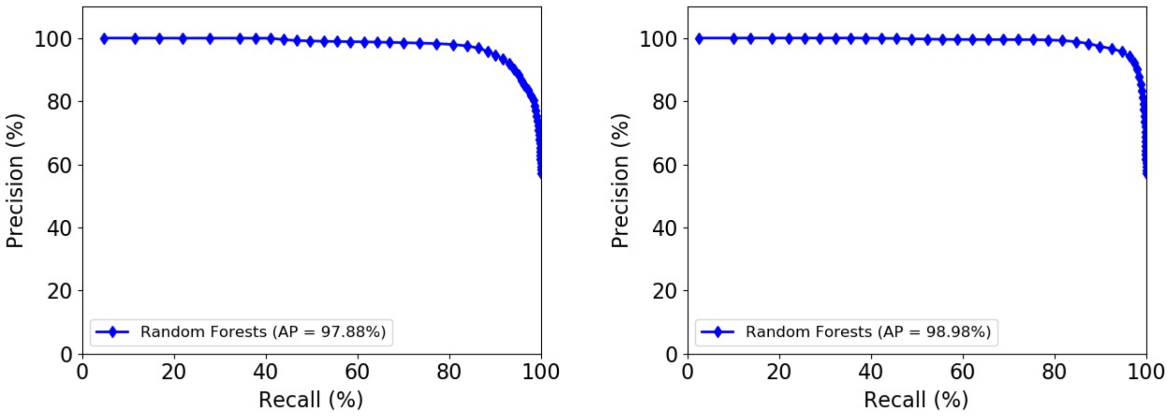

(a) Outer Cross-validation

(b) Test

Figure 7. Precision-Recall curves for Nonwear

6 Nap classification

The data points corresponding to the comparison between self-reported habitual nap duration per week and accelerometer-based estimates as discussed in the main paper are shown in Figure 17.

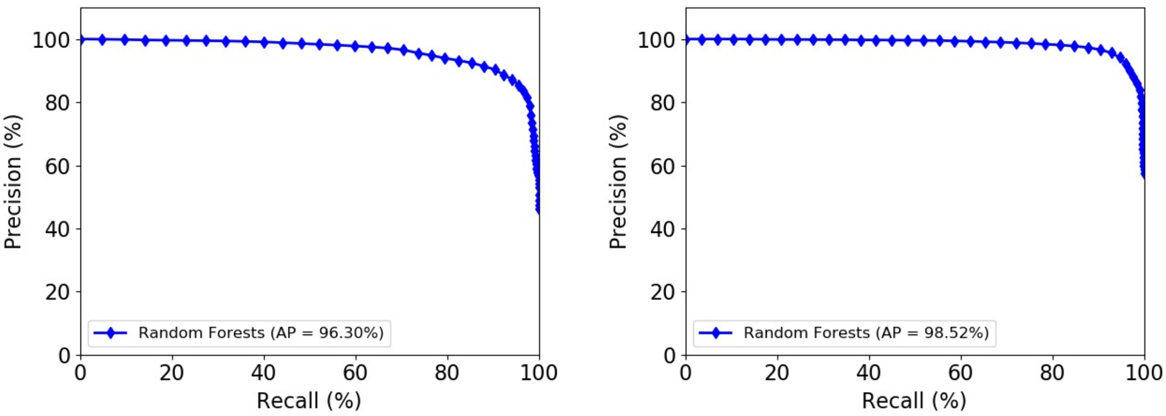

(a) Outer Cross-validation

(b) Test

Figure 8. Precision-Recall curves for Wear

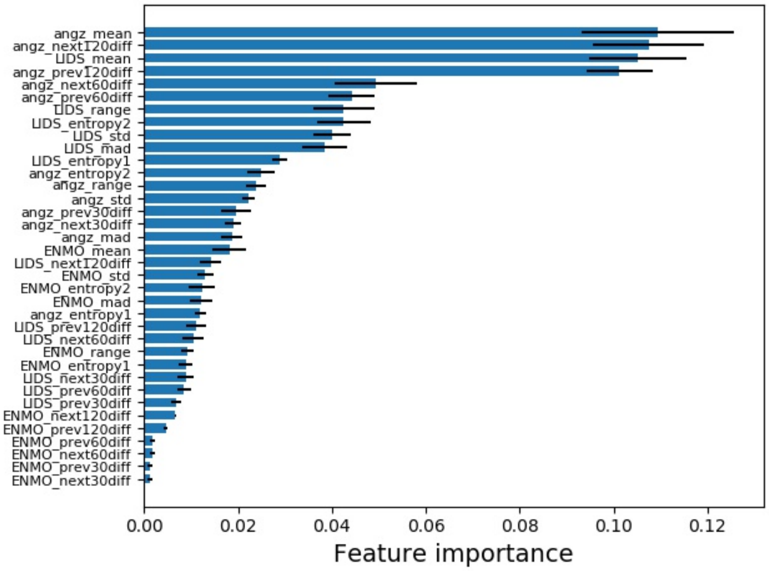

Figure 9. Random Forests feature importances for nonwear classification

7 Sleep-Wake classification using Deep learning

We investigated sleep classification with learned features from raw data using a convolutional residual neural network[12]. Raw accelerometer signals (a_x, a_y, a_z) and the three derived feature signals, ENMO, Z-angle and LIDS, were used as input feature channels. Each sample spans a time interval of 30 seconds and the signals were resampled to 50Hz before being fed to the neural network.

The residual network shown in Figure 18 starts with a 1-dimensional convolution layer with 32 7×1 filters and a stride of 2. This was followed by a convolutional residual block and two identity residual blocks with 16,16 and 32 filters of size 3×1 each. The output of the residual blocks was fed to a 1-dimensional max-pooling layer. It was followed by a fully connected layer and a softmax classification layer.

The network layers were initialized using Glorot uniform initializer. The network parameters were constrained using a max norm constraint to prevent overfitting. LeakyReLU activations were used throughout the network with $\alpha = 0.1$. We used batch renormalization layers[13] since our data is not *i.i.d*, *i.e.* time series data split up into samples spanning 30 seconds each. The network was trained with renormalization turned off $(r = 1, d = 0)$ for the first epoch and momentum = 0.9. From second epoch, r was gradually increased to 2 and d was increased to 4 over the next two epochs.

Table 6. Sleep stage classification
F1 - F1-score, AP - Average Precision

Dataset	F1 (%)	AP (%)
Outer Cross-validation	33.53	37.59
Test	34.49	36.58

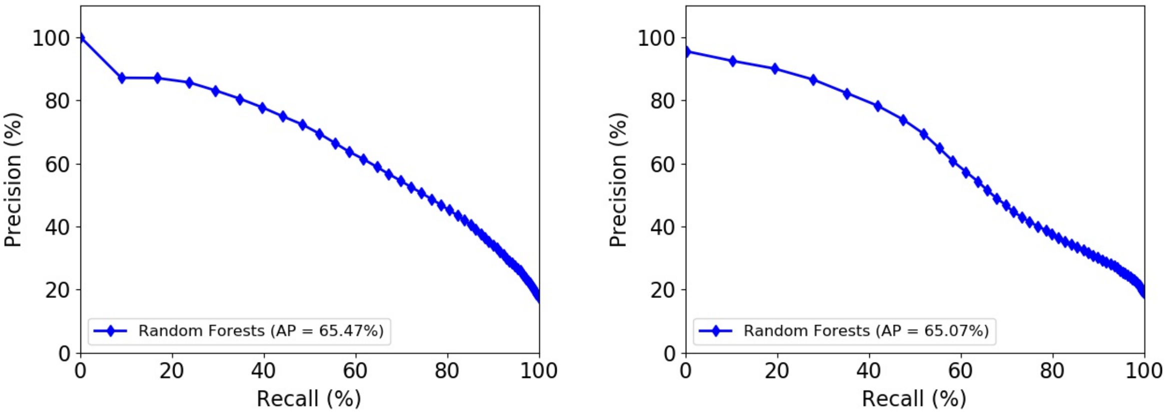

(a) Outer Cross-validation

(b) Test

Figure 10. Sleep stage classification: Precision-Recall curves for Wake

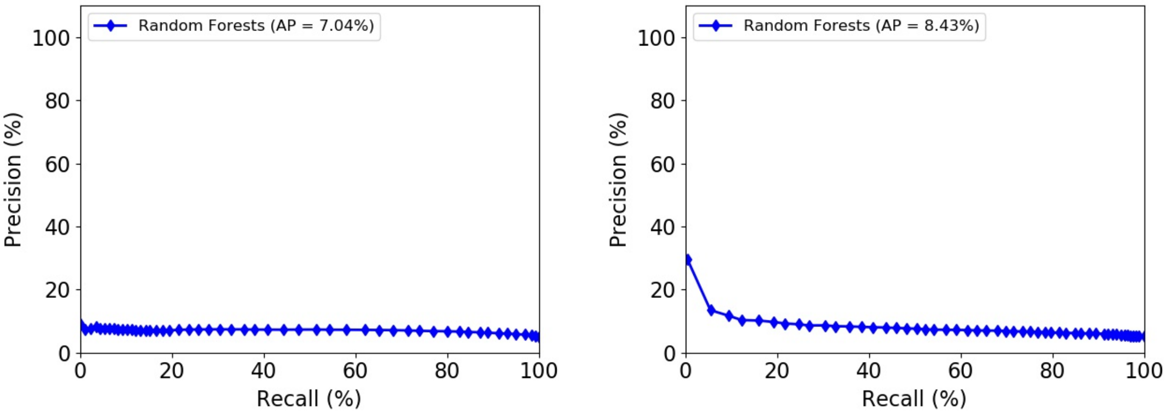

(a) Outer Cross-validation

(b) Test

Figure 11. Sleep stage classification: Precision-Recall curves for NREM 1

The network was optimized using Adam optimization algorithm[14]. Instead of the cross-entropy loss functions, we used the custom focal loss function as our objective function since it has been shown to be effective for highly imbalanced scenarios like object detection[15]. Let p be the estimated probability of a sample belonging to class with label $y = 1$. Let

$$p_t = \begin{cases} p, & \text{if } y = 1 \\ 1 - p, & \text{otherwise} \end{cases} \quad (1)$$

The focal loss is given by

$$FL(p_t) = -\alpha_t (1 - p_t)^{\gamma} log(p_t) \quad (2)$$

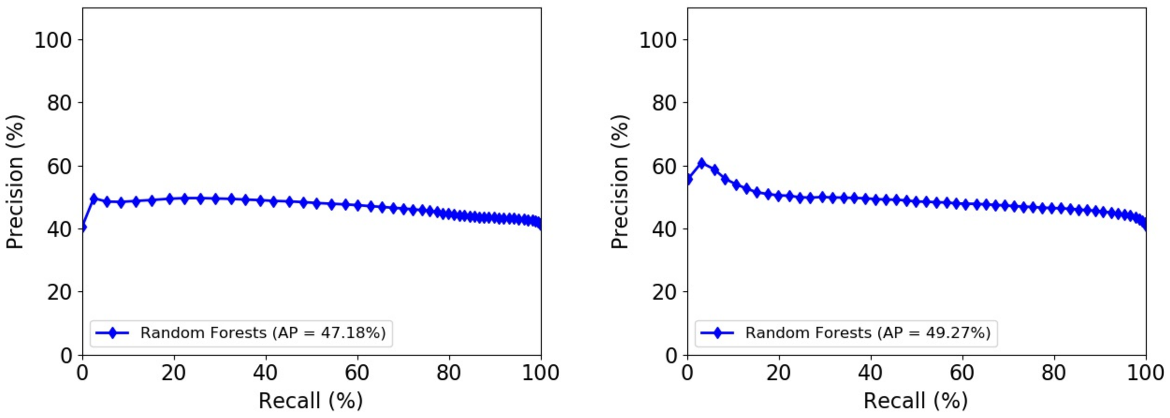

(a) Outer Cross-validation

(b) Test

Figure 12. Sleep stage classification: Precision-Recall curves for NREM 2

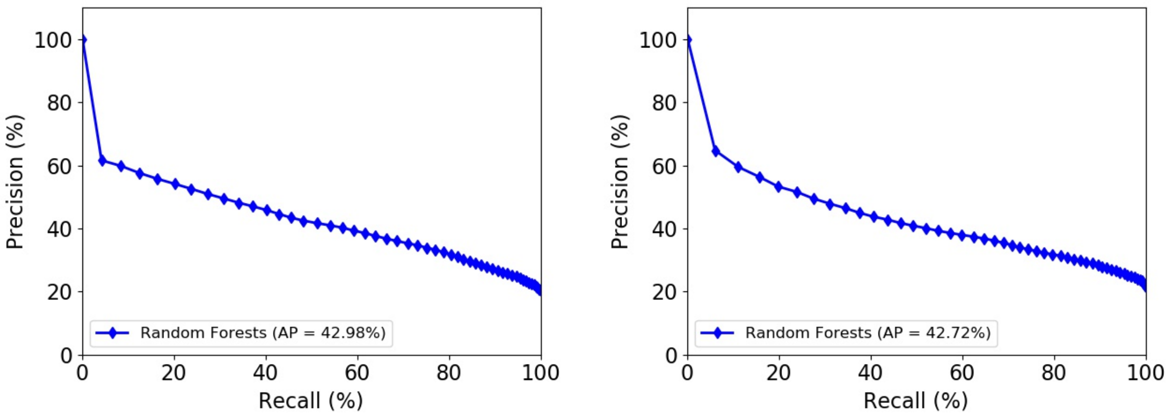

(a) Outer Cross-validation

(b) Test

Figure 13. Sleep stage classification: Precision-Recall curves for NREM 3

where $\alpha \in [0,1]$ is the weighting factor for classes and $\gamma \geq 0$ is the focusing factor. The focusing factor increases the loss for samples with low predicted probabilities (*i.e.* hard examples) thereby making the model focus more on hard samples. In our experiments, we used $\gamma = 2.0$ and $\alpha = 1.0$.

We used a five-fold nested cross-validation approach to determine sleep classification performance using Resnets. Similar to the dataset partitioning described in the main paper, in the outer fold, the data is split into training and test partitions such that users in both partitions do not overlap in each fold. The training partition is further divided into training and validation data (4:1) such that the validation data is used to chose the optimal hyperparameters and best trained model. The tuned hyperparameters include the learning rate, maxnorm constraint,dropout rate and the number of hidden units in the penultimate dense layer. The model was trained with a batch size of 64 for 50 epochs. During training, we used data augmentation to make our model robust to various perturbations. Transformations such as jitter, time warping, accelerometer rotation and random sampling were used to augment the data. During training, the samples were shuffled every epoch and each minibatch was balanced to have similar number of samples from both classes.

Resnets were trained and evaluated using only the Amsterdam dataset. The van Hees approach and Random Forests approach were used to determine the effectiveness of Resnets using the same dataset. The classification performance metrics across the five outer folds are averaged and reported in Table 7. The precision-recall curves for Wake and Sleep classes are shown in Figure 19.

It can be seen that Random Forests approach performs better than the heuristic vanHees approach and Resnets. Specifically,

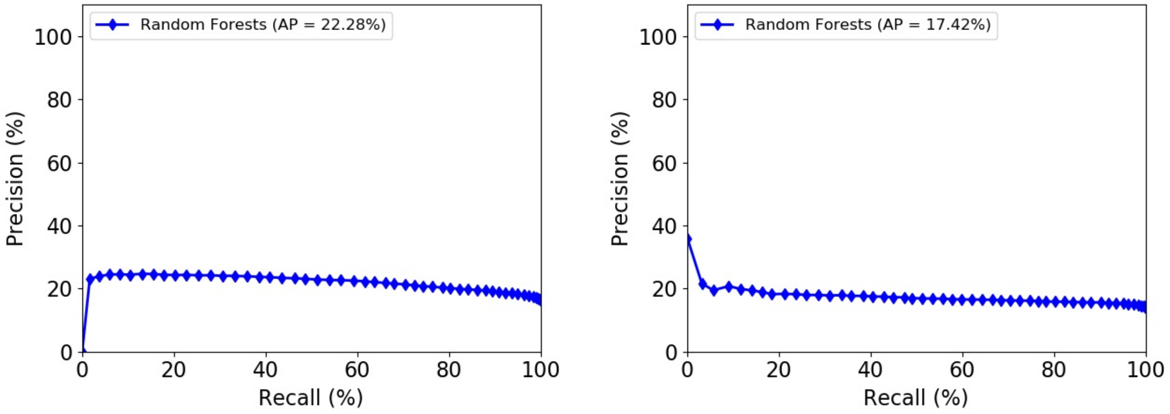

(a) Outer Cross-validation (b) Test

Figure 14. Sleep stage classification: Precision-Recall curves for REM

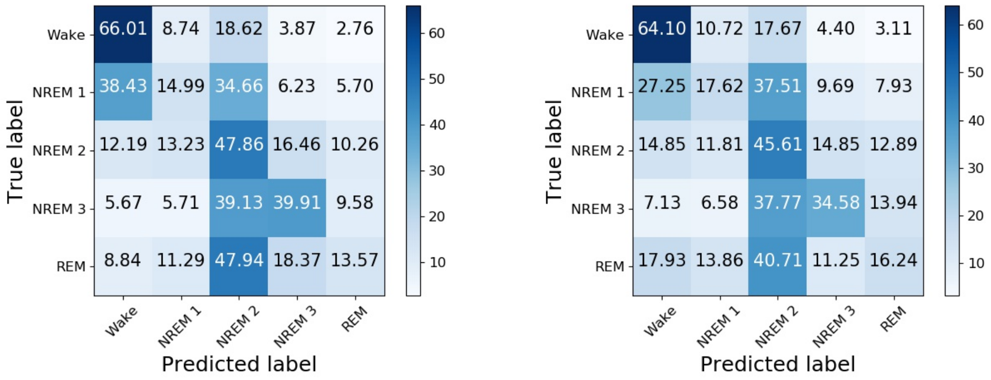

(a) Outer Cross-validation (b) Test

Figure 15. Confusion matrices for Sleep stage classification

the classification performance of all three approaches are similar for the Sleep class whereas Random Forests perform better at Wake prediction than other two approaches. Resnets seem to struggle with Wake prediction for the Amsterdam dataset in which Wake samples constitute only 14% of the data. In spite of balancing classes with data augmentation during training, Resnets fail to be effective for Wake prediction.

A possible explanation for the poor performance of our deep learning approach is the use of a 30 second window, by which only signal features shorter than 30 seconds can be learnt. The choice for a 30 second window was driven by computational constraints. The larger the window size the longer the training process. A more powerful computational infrastructure capable of handling larger time windows is desirable.

References

1. R Core Team. *R: A Language and Environment for Statistical Computing*. R Foundation for Statistical Computing, Vienna, Austria (2020).
2. van Hees, V. T., Fang, Z., Zhao, J. H. & Heywood, J. Package ggir. *CRAN* (2019).
3. Migueles, J. H., Rowlands, A. V., Huber, F., Sabia, S. & van Hees, V. T. GGIR: A Research Community–Driven Open Source R Package for Generating Physical Activity and Sleep Outcomes From Multi-Day Raw Accelerometer Data. *J. for Meas. Phys. Behav.* **2**, 188–196, DOI: 10.1123/jmpb.2018-0063 (2019).
4. Sundararajan, K. Code repository for the work presented in this paper. https://github.com/wadpac/SleepStageClassification.

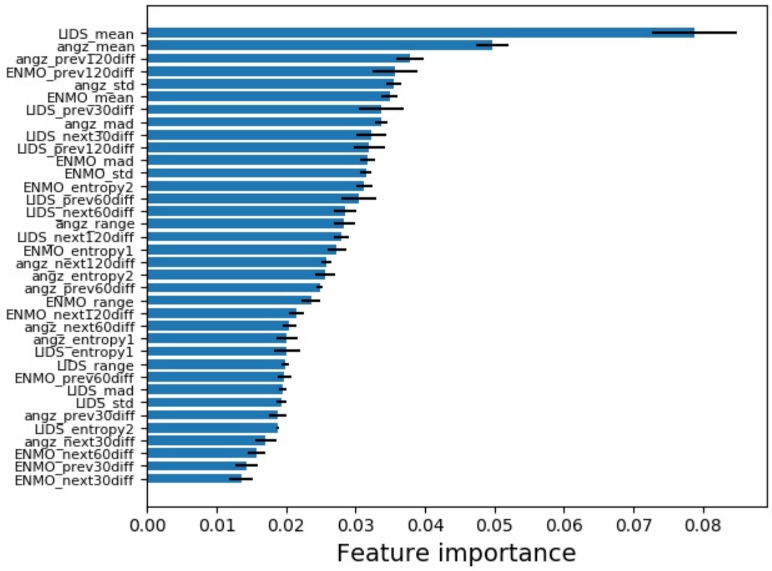

Figure 16. Random Forests feature importances for sleep stage classification

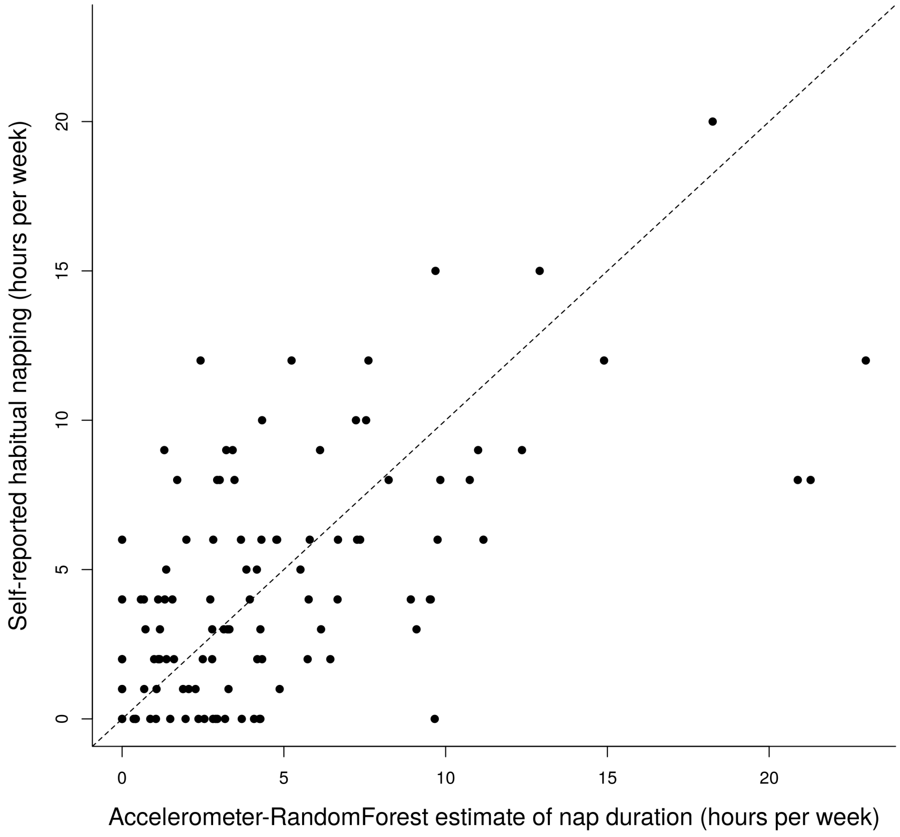

Figure 17. Comparison self-reported and accelerometer-based estimate of nap duration (r=0.60, p < .001). Dashed line: line of identity.

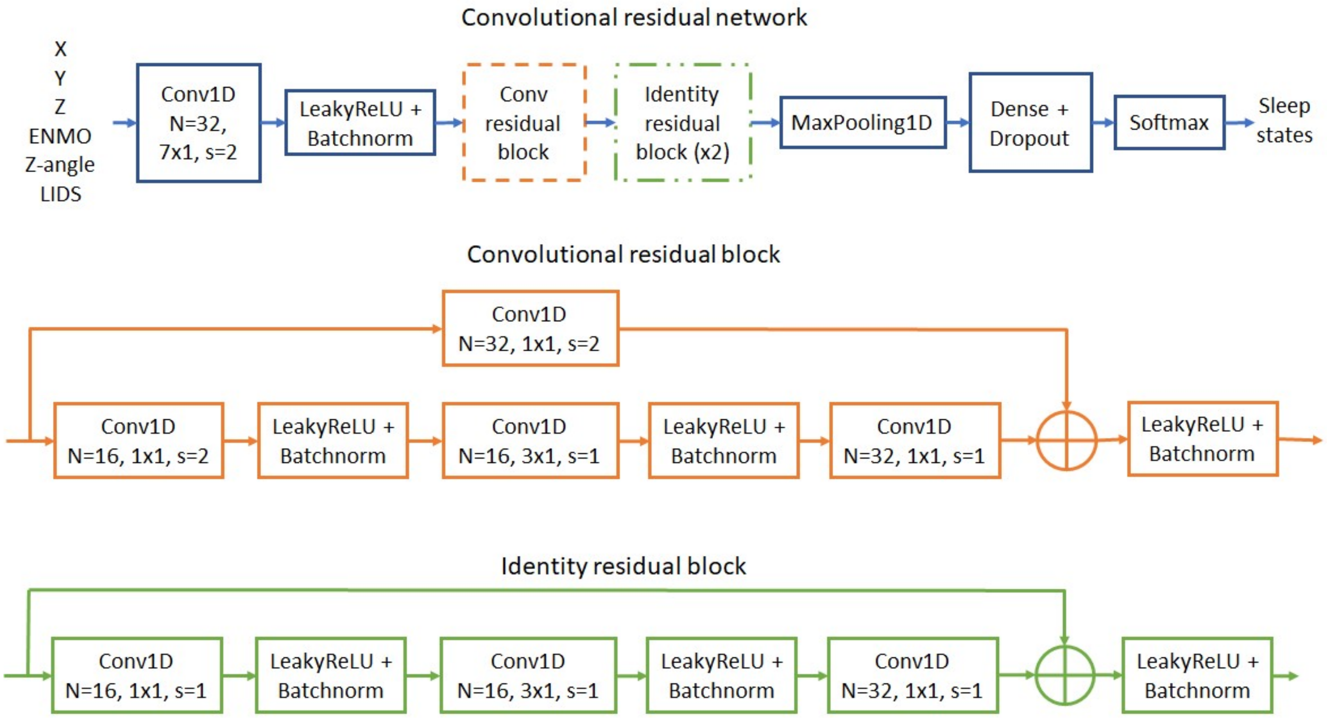

Figure 18. Residual network for sleep stage classification

Table 7. Sleep-Wake classification
F1 - F1-score, AP - Average Precision

Approach	Amsterdam	
	F1 (%)	AP (%)
vanHees	70.37	67.51
Random Forests	73.01	76.78
Resnets	68.32	72.09

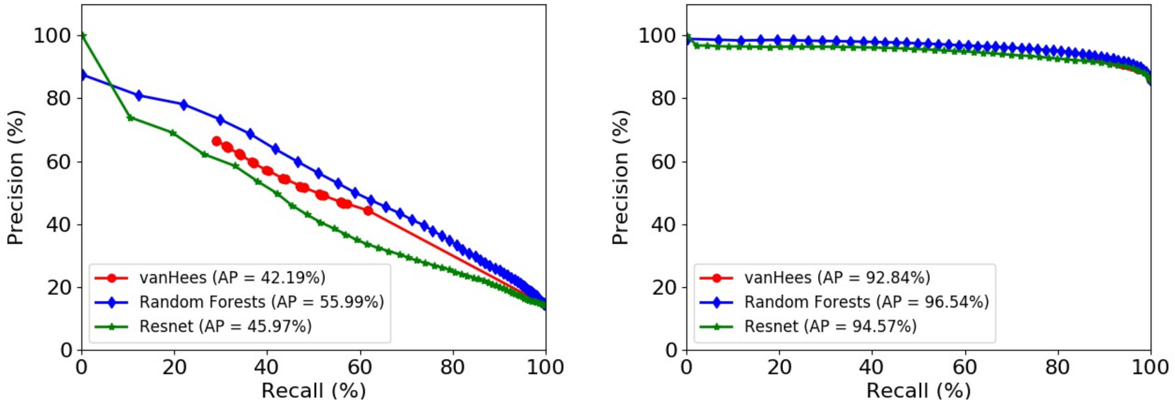

(a) Wake (b) Sleep

Figure 19. Sleep-Wake classification: Precision-Recall curves using Resnets

5. Van Hees, V. T. *et al.* Autocalibration of accelerometer data for free-living physical activity assessment using local gravity and temperature: an evaluation on four continents. *J. Appl. Physiol.* **117**, 738–744 (2014).

6. van Hees, C. S., V.T. & Anderson, K. Newcastle polysomnography and accelerometer data. *Zenodo.org.* DOI: 10.5281/zenodo.1160410 (2018).

7. van Hees, V. T. *et al.* A Novel, Open Access Method to Assess Sleep Duration Using a Wrist-Worn Accelerometer. *PloS one* **10**, e0142533, DOI: 10.1371/journal.pone.0142533 (2015).

8. Byrne, E. M., Gehrman, P. R., Trzaskowski, M., Tiemeier, H. & Pack, A. I. Genetic correlation analysis suggests association between increased self-reported sleep duration in adults and schizophrenia and type 2 diabetes. *Sleep* **39**, 1853–1857 (2016).

9. van Hees, V. T. *et al.* Estimating sleep parameters using an accelerometer without sleep diary. *Sci. reports* **8**, 12975 (2018).

10. Te Lindert, B. T. F. v. d. M. W. P. D. K. W. R. v. d. W. Y. D. R. J. R. . V. S. E., B. Actigraphic multi-night home-recorded sleep estimates reveal three types of sleep misperception in Insomnia Disorder and good sleepers. *J. sleep research* **1**, e12937, DOI: 10.1111/jsr.12937 (2020).

11. Stress and health survey. https://www.ucl.ac.uk/epidemiology-health-care/sites/epidemiology-health-care/files/s11-questionnaire.pdf.

12. He, K., Zhang, X., Ren, S. & Sun, J. Deep residual learning for image recognition. In *Proceedings of the IEEE conference on computer vision and pattern recognition*, 770–778 (2016).

13. Ioffe, S. Batch renormalization: Towards reducing minibatch dependence in batch-normalized models. In *Advances in neural information processing systems*, 1945–1953 (2017).

14. Kingma, D. P. & Ba, J. Adam: A method for stochastic optimization. *arXiv preprint arXiv:1412.6980* (2014).

15. Lin, T.-Y., Goyal, P., Girshick, R., He, K. & Dollár, P. Focal loss for dense object detection. In *Proceedings of the IEEE international conference on computer vision*, 2980–2988 (2017).
